# Combined Network Pharmacology, Transcriptomics and Metabolomics Strategies Reveal the Mechanism of Action of Lang Chuang Wan to Ameliorate Lupus Nephritis in MRL/lpr Mice

**DOI:** 10.3390/ph18060916

**Published:** 2025-06-18

**Authors:** Cuicui Li, Guoxin Ji, Xinru Zhang, Hang Yu, Zhimeng Li, Bo Yang, Zhuangzhuang Yao, Shilei Wang, Tongwei Jiang, Shumin Wang

**Affiliations:** 1 College of Integrated Chinese and Western Medicine, Changchun University of Chinese Medicine, Changchun 130117, China; 18844168431@163.com; 2 College of Pharmacy, Changchun University of Chinese Medicine, Changchun 130117, China; 17843099892@163.com (G.J.); zhangxr99911@163.com (X.Z.); lzm2084@163.com (Z.L.); yangbo0623@126.com (B.Y.); 13461743039@163.com (Z.Y.); 3 College of Traditional Chinese Medicine, Changchun University of Chinese Medicine, Changchun 130117, China; yuhang202406@163.com (H.Y.); xlxzwsl@163.com (S.W.)

**Keywords:** lupus nephritis, network pharmacology, transcriptomics, metabolomics, PI3K/AKT/mTOR signaling pathway

## Abstract

**Background**: Lupus nephritis (LN) is a serious complication of systemic lupus erythematosus (SLE) and is difficult to cure. Lang Chuang Wan (LCW) has been widely used in clinical practice as a treatment for SLE and LN, but its active ingredients and mechanism of action have not been elucidated. To address this, we aim to analyze LCW’s chemical components and clarify its mechanisms in treating LN. **Methods**: We utilized ultra-performance liquid chromatography–tandem mass spectrometry (UPLC-MS/MS) to analyze the components of LCW and assessed its effects on MRL/lpr mice through ELISA, H&E staining, Masson’s trichrome staining, and IgG immunofluorescence. Then, we further explored the mechanisms of action through network pharmacology, transcriptomics, and metabolomics, and validated with Western blot. **Results**: LCW contained 1303 chemical components, primarily flavonoids and terpenoids. It significantly improved kidney pathology and normalized levels of serum ANA, anti-dsDNA, anti-Sm, C3, C4, Cr, BUN, IL-6, IL-10, IL-17, TNF-α, and urinary protein (UP) in MRL/lpr mice. Network pharmacology, transcriptomics, and metabolomics indicated that LCW’s therapeutic effect on LN involved the PI3K/AKT pathway, confirmed by Western blot showing LCW’s suppression of the PI3K/AKT/mTOR pathway. **Conclusions**: LCW alleviates pathological symptoms in MRL/lpr mice by inhibiting the PI3K/AKT/mTOR signaling pathway, providing insights into its therapeutic mechanisms for lupus nephritis.

## 1. Introduction

Systemic lupus erythematosus (SLE) is a chronic autoimmune disease that can lead to damage across various organs in the body [1]. Lupus nephritis (LN) is a prevalent and serious complication of SLE, impacting around 30 to 40% of individuals diagnosed with the disease [2]. LN primarily manifests as glomerular damage, characterized by inflammation, cell proliferation, necrosis, and injury to podocytes and tubular epithelial cells [3]. The disease has a high recurrence rate, severely impacting patients’ quality of life [4]. Its pathogenesis involves a complex interplay of genetic, epigenetic, hormonal, and other factors [5]. LN treatment is typically divided into two phases: the initial phase and the maintenance phase [6], with corticosteroids, antimalarials, immunosuppressants, and biologics being the primary therapies [7]. While these treatments are effective, their side effects cannot be overlooked. More critically, despite aggressive treatment, some patients may still progress to end-stage renal disease (ESRD) [8]. This highlights the limitations of current treatment strategies and underscores the urgent need for safer, more effective alternative or adjunct therapies.

Traditional Chinese medicine (TCM) has shown potential in treating autoimmune diseases due to its unique characteristics, including multi-component, multi-target, and bidirectional regulatory properties [9]. Lang Chuang Wan (LCW), a compound formula containing 16 TCM ingredients, has been part of the Chinese pharmacopeia since 2005. LCW is known for its heat-clearing, detoxifying, blood-cooling, and circulation-promoting effects and has demonstrated promising efficacy in treating SLE. Existing studies indicate that LCW modulates inflammation in resiquimod-induced RAW264.7 mouse cells [10]. Research by Guoxin Ji and colleagues have found that LCW significantly reduced urinary protein (UP) levels, anti-double-stranded DNA antibodies (anti-dsDNA), anti-nuclear antibodies (ANA), and inflammatory cytokines (TNF-α, IL-6) in a mouse model of SLE-like disease [11]. These results suggest that LCW may alleviate inflammation, regulate immune responses, and protect kidney function, although its underlying mechanisms warrant further investigation.

In recent years, network pharmacology has emerged as a crucial tool for uncovering the mechanisms underlying the actions of TCM [12]. Additionally, the rapid advancement of high-throughput technologies, such as metabolomics, has enabled the identification of biological processes that have already occurred [13], playing a crucial role in autoimmune disease research [14]. Transcriptomics, with its capacity for quantitative analysis and efficient screening, can identify and quantify thousands of differentially expressed genes [15], providing a powerful approach for exploring the complex pathological mechanisms of LN [16]. These advanced technologies offer new perspectives and strategies for modern research on LCW. In this study, we utilize ultra-performance liquid chromatography–tandem mass spectrometry (UPLC-MS/MS) to identify the major active components of LCW. Enzyme-linked immunosorbent assay (ELISA) is employed to assess the therapeutic effects of LCW in treating LN. Histopathological analysis using H&E staining, Masson’s trichrome staining, and immunofluorescence (IF) is conducted to observe renal improvements in MRL/lpr mice treated with LCW. By integrating visual analysis from network pharmacology, transcriptomics, and metabolomics, we predict the potential mechanisms of LCW in treating LN. Finally, Western blot is used to validate these mechanisms, clarifying LCW’s specific role in regulating the expression of key proteins.

## 2. Results

### 2.1. Active Components of LCW

**Figure 1:**
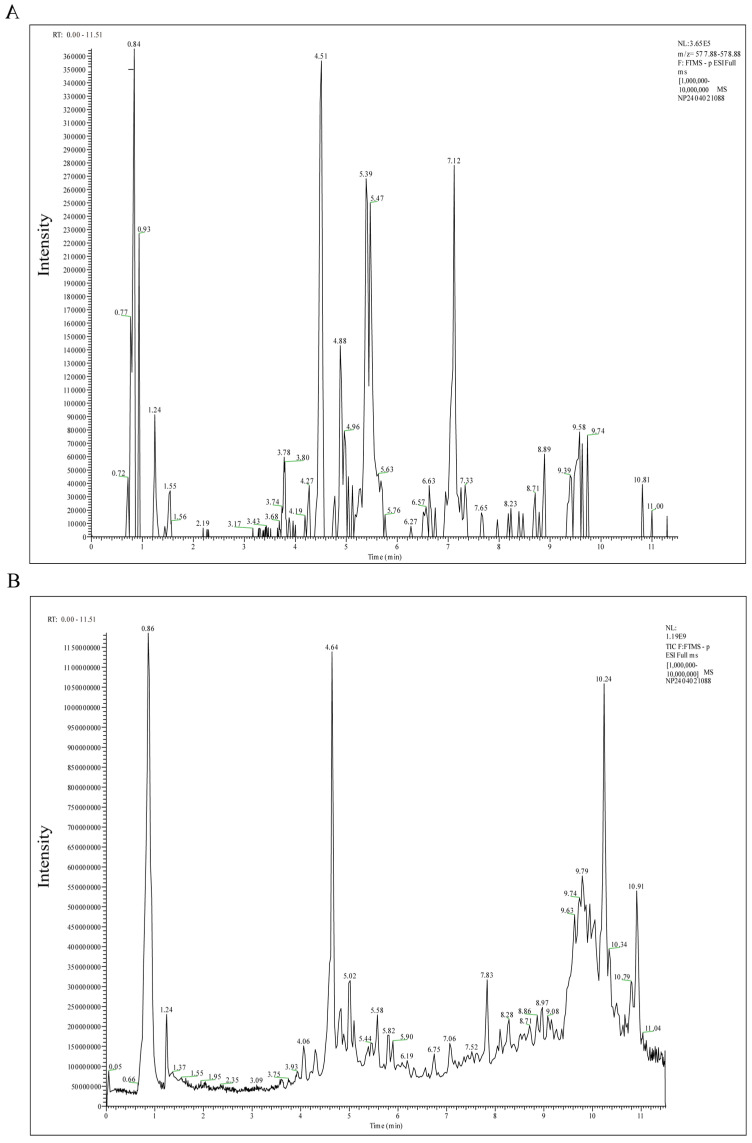
UPLC-MS/MS analysis of LCW. (**A**) Positive ion base peak chromatogram of LCW based on UPLC-MS/MS. (**B**) Negative ion base peak chromatogram of LCW based on UPLC-MS/MS.

Using UPLC-MS/MS technology, 1303 metabolites were identified in LCW, and their base peak chromatogram (BPC) is shown in Figure 1. The top 25 active components are listed in Table 1. The active components of LCW are predominantly natural compounds, including flavonoids, terpenoids, and phenolic acids.

### 2.2. LCW Regulates the Levels of Serum Autoantibodies and Inflammatory Cytokines in MRL/lpr Mice, Enhancing Renal Function and Reducing Kidney Damage

**Figure 2:**
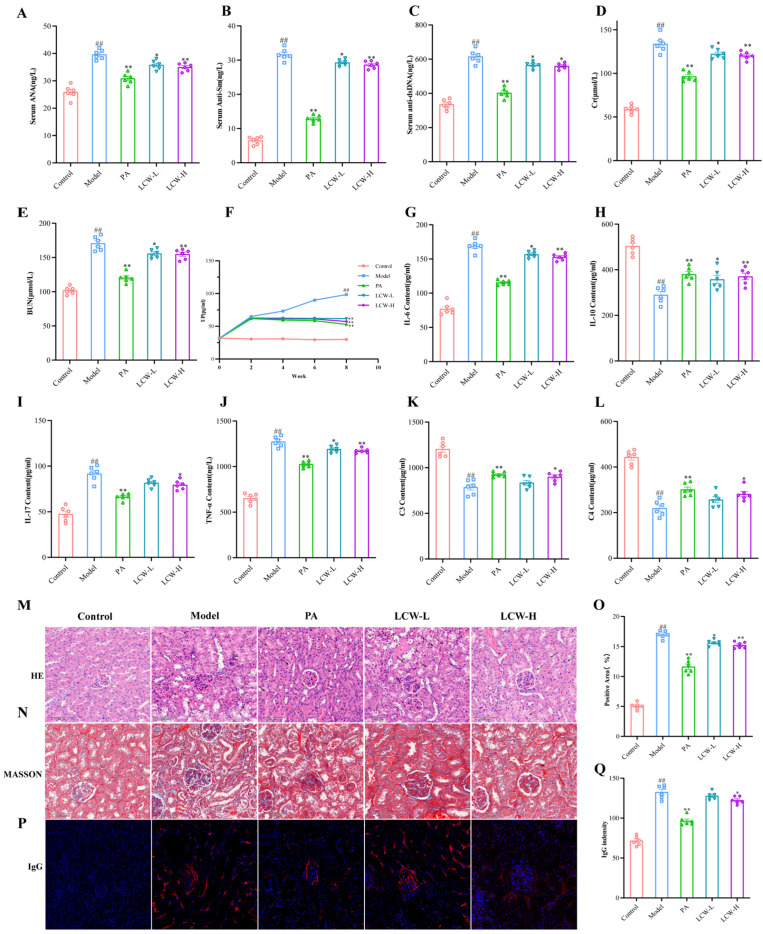
Comprehensive analysis of serum autoantibody levels, renal function, inflammatory cytokine levels, and renal histopathology. The evaluated biomarkers include the following: (**A**) ANA, (**B**) anti-dsDNA, (**C**) anti-Sm, (**D**) Cr, (**E**) BUN, (**F**) UP, (**G**) IL-6, (**H**) IL-10, (**I**) IL-17, (**J**) TNF-α, (**K**) C3, and (**L**) C4. Histopathological assessment includes the following: (**M**) H&E staining results, with black arrows indicating tubular epithelial cell edema with deformation and necrosis, and white arrows indicating interstitial inflammatory cell infiltration. (**N**) Masson’s trichrome staining results. (**O**) Quantification of renal fibrosis scoring. (**P**) Results of IgG immune complex deposition. (**Q**) Quantification of IgG immune complex deposition. (Magnification 40×, Scale bar = 100 μm.) Data are presented as mean ± standard error of the mean (SEM), ^##^ *p* < 0.01 indicate statistically significant differences compared to the control group; * *p* < 0.05 and ** *p* < 0.01 indicate statistically significant differences compared to the model group.

The ELISA test results (Figure 2A–L) indicate that, compared to the control group, the levels of ANA, anti-dsDNA, and anti-Sm were significantly elevated in the model group (*p* < 0.01). Following treatment, antibody titers in the PA group, LCW-L group, and LCW-H group showed significant reductions, with *p* < 0.01, *p* < 0.05, and *p* < 0.01, respectively. Regarding renal function markers, the levels of Cr, BUN, and UP in the model group were significantly higher than those in the control group (*p* < 0.01). After treatment with PA and LCW, these levels were significantly restored. Specifically, in the PA and LCW-H groups, Cr, BUN, and UP levels were significantly reduced (*p* < 0.01). The LCW-L group also showed significant reductions in Cr, BUN, and UP levels (*p* < 0.05, *p* < 0.05, *p* < 0.01, respectively). For inflammation markers, compared to the control group, the levels of IL-6, IL-17, and TNF-α in the model group were significantly elevated (*p* < 0.01), while IL-10 was significantly decreased (*p* < 0.01). After treatment, the levels of inflammatory cytokines in the PA group were significantly improved (*p* < 0.01). In the LCW-L group, although IL-17 levels did not show a significant change, IL-6 and TNF-α levels were significantly reduced (*p* < 0.05), and IL-10 showed a trend towards increased levels (*p* < 0.05). In the LCW-H group, all tested cytokines IL-17 (*p* < 0.05), IL-6 (*p* < 0.01), and TNF-α (*p* < 0.01) were significantly reduced, while IL-10 was significantly elevated (*p* < 0.01). Additionally, the levels of complement components C3 and C4 in the model group were significantly lower than those in the control group (*p* < 0.05), with a more significant reduction in C4 (*p* < 0.01). Following treatment, C3 and C4 levels in the PA group were significantly increased (*p* < 0.01). In the LCW-H group, both C3 and C4 levels were significantly elevated (*p* < 0.05). However, the LCW-L group did not significantly affect the levels of C3 and C4.

H&E staining results (Figure 2M) show that the glomeruli in the control group are intact, with no abnormal pathological structures observed. In contrast, the model group mice exhibited glomerular capillary hyperplasia, increased glomerular volume, reduced Bowman’s space, focal tubular epithelial cell edema with deformation and necrosis, and tubular lumen dilation. Compared to the model group, the severity of renal lesions in the PA, LCW-L, and LCW-H groups was significantly reduced. Masson’s staining results (Figure 2N,O) revealed that, compared to the control group, the area of renal interstitial fibrosis in the model group was significantly increased (*p* < 0.01). After treatment, the area of renal interstitial fibrosis in the PA, LCW-L, and LCW-H groups was significantly reduced (*p* < 0.01, *p* < 0.05, *p* < 0.01, respectively). IF results (Figure 2P,Q) showed that, compared to the control group, the deposition of IgG immune complexes in the model group was significantly increased (*p* < 0.01). After treatment, IgG immune complex deposition in the PA and LCW-H groups was significantly reduced (*p* < 0.01 and *p* < 0.05, respectively). Conversely, while the LCW-L group showed a decrease in IgG deposition, this change was not statistically significant.

Combining the serum biomarker data with the pathological analysis results, LCW-H demonstrated more significant effects in regulating immunity, exerting anti-inflammatory effects, improving renal function, and inhibiting fibrosis, when compared to LCW-L. Based on these findings, subsequent analyses will focus on exploring the mechanisms and pathways through which LCW-H exerts its effects.

### 2.3. Network Pharmacology Prediction for LCW Treatment of LN

**Figure 3:**
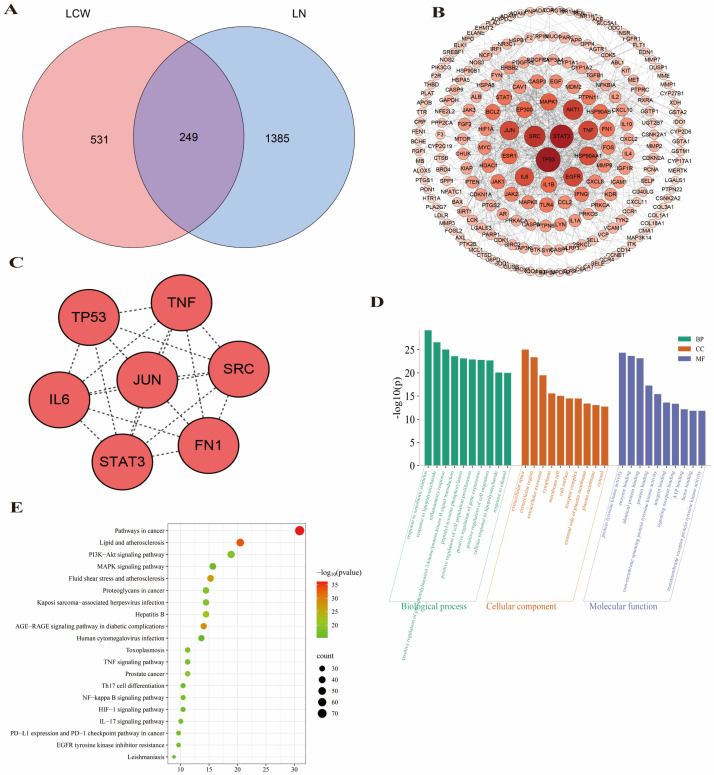
Network pharmacology prediction of the LCW treatment for LN. (**A**) Venn diagram of the overlapping targets between LCW and LN. (**B**) PPI network of LCW acting on 249 common targets. (**C**) Potential core targets of LCW action. (**D**) GO functional enrichment analysis of LCW treatment targets for LN. (**E**) KEGG pathway enrichment analysis of LCW treatment targets for LN.

A total of 965 potential targets for the active components of LCW and 1634 targets related to LN were identified. Among these two sets, 249 common targets were found (Figure 3A). These overlapping targets were imported into the STRING database and analyzed using Cytoscape 3.7.2 software to construct a PPI network (Figure 3B). The network contained 248 nodes and 2453 edges. Based on an analysis of network parameters including degree, betweenness centrality, and closeness centrality seven key targets were identified (Figure 3C). GO analysis (Figure 3D) revealed that the biological processes (BP) most commonly associated with these targets included responses to xenobiotic stimuli, lipopolysaccharide, and inflammation. Cellular components (CC) were predominantly located in extracellular exosomes, cytoplasm, and the plasma membrane. Molecular functions (MF) primarily involved enzyme binding, protein binding, and signaling receptor binding. The KEGG pathway analysis (Figure 3E) indicated that the LCW treatment for LN potentially influences several critical signaling pathways, such as the PI3K-AKT pathway, Mitogen-Activated Protein Kinase (MAPK) pathway, and the Nuclear Factor Kappa B (NF-κB) pathway.

### 2.4. Transcriptomics Analysis Results

**Figure 4:**
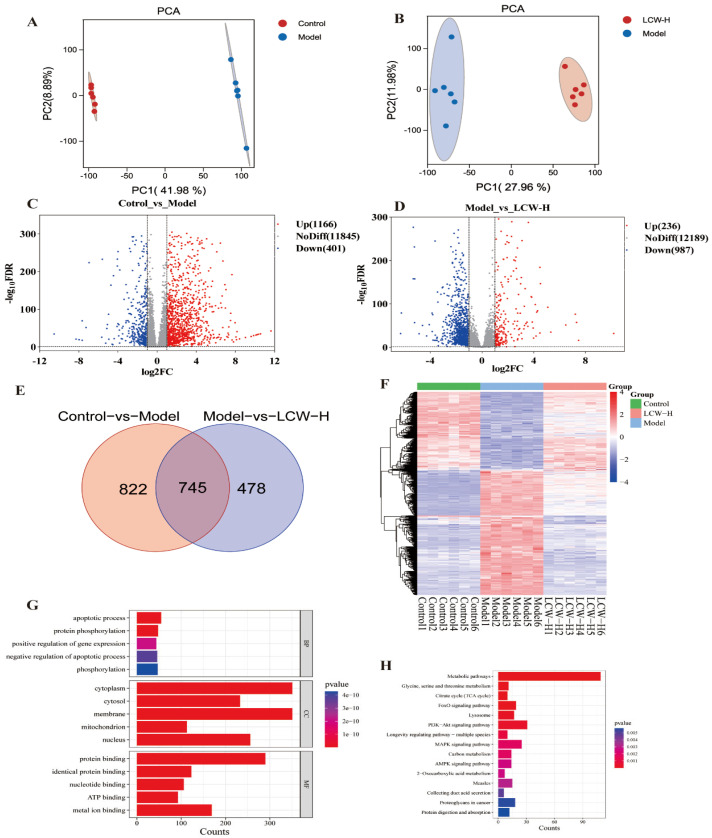
Transcriptomics analysis results (*n* = 6). (**A**) PCA plot of control and model group samples. (**B**) PCA plot of model and LCW-H group samples. (**C**) Volcano plot of DEGs between control and model groups. (**D**) Volcano plot of DEGs between model and LCW-H groups. (**E**) Venn diagram of overlapping DEGs between control-vs.-model and model-vs.-LCW-H comparison groups. (**F**) Heatmap of hierarchical clustering analysis of DEGs. (**G**) GO analysis results of DEGs. (**H**) KEGG analysis results of DEGs.

RNA sequencing (RNA-seq) analysis was performed on the transcripts from the control, model, and LCW-H groups for comparison, generating a total of 114.74 Gb of clean data. Each sample’s clean data reached 5.68 Gb, with a Q30 base percentage of 93.15% or higher. The clean reads from each sample were then mapped to the designated reference genome, with alignment efficiency varying between 95.88% and 97.31%, demonstrating the high quality of the transcriptomic sequencing. PCA score plots (Figure 4A,B) revealed clear separation between the control and model groups, as well as between the model and LCW-H groups. Figure 4C–F display the DEGs between the control and model groups, and between the model and LCW-H groups, respectively. In comparison to the control group, the model group exhibited 1166 genes that were upregulated and 401 genes that were downregulated (Figure 4C). In contrast, the LCW-H group exhibited 236 upregulated genes and 987 downregulated genes relative to the model group (Figure 4D). A comparison of the two transcriptomic datasets identified 745 overlapping DEGs (Figure 4E). Figure 4F presents the hierarchical clustering analysis of DEGs, illustrating expression changes in the model group that were subsequently reversed by LCW treatment. Further Gene Ontology (GO) analysis of the DEGs, shown in Figure 4G, revealed that BP were predominantly associated with key pathways such as apoptosis, protein phosphorylation, and the positive regulation of gene expression. In terms of CC, these processes primarily occurred in essential structures like the cytoplasm, cell membrane, and mitochondria. MF analysis highlighted important functions, including protein binding, nucleotide binding, and ATP binding. KEGG pathway analysis (Figure 4H) revealed notable enrichment of the DEGs in several key pathways, including the FOXO signaling pathway, PI3K-AKT signaling pathway, and MAPK signaling pathway.

### 2.5. Results of Metabolomics Analysis

**Figure 5:**
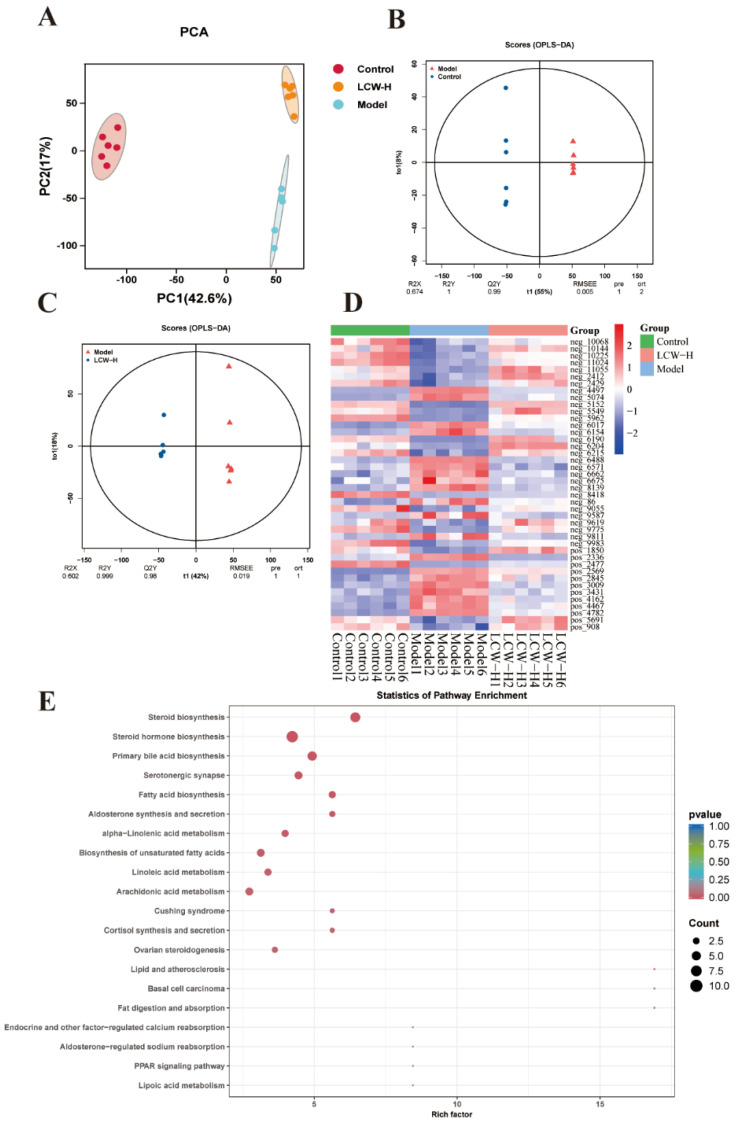
Metabolomics analysis results (*n* = 6). (**A**) PCA of control, model, and LCW-H groups. (**B**) OPLS-DA analysis between control and model groups. (**C**) OPLS-DA analysis between model and LCW-H groups. (**D**) Heatmap visualization of metabolic biomarkers in control, model, and LCW-H groups. (**E**) Enrichment analysis of candidate biomarkers.

Metabolomics data from the control, model, and LCW-H groups were analyzed using multivariate statistical methods with an LC/MS system. PCA was conducted to offer a comprehensive overview of the data, identify potential outliers, and evaluate the variations in serum metabolites across the three groups. Figure 5A clearly illustrates the significant differences in metabolic profiles between the control, model, and LCW-H groups. The OPLS-DA score plots (Figure 5B,C) highlight the intergroup differences between control vs. model and model vs. LCW-H, with VIP values reported. These results suggest that LCW intervention effectively ameliorated the metabolic disturbances in MRL/lpr mice. Through screening, 42 biomarkers closely associated with lipid metabolism were identified, which exhibited significant changes following LCW-H treatment (Table 2). Heatmap analysis (Figure 5D) further underscores the significant impact of LCW-H on the metabolic characteristics of MRL/lpr mice, revealing its prominent regulatory effect. Using the BMKCloud platform for analysis of these candidate biomarkers (Figure 5E) showed significant enrichment in metabolic pathways, including steroid biosynthesis, steroid hormone biosynthesis, and arachidonic acid metabolism (*p* < 0.05).

### 2.6. Integrated Multi-Omics Analysis of LCW Treatment in MRL/lpr Mice

**Figure 6:**
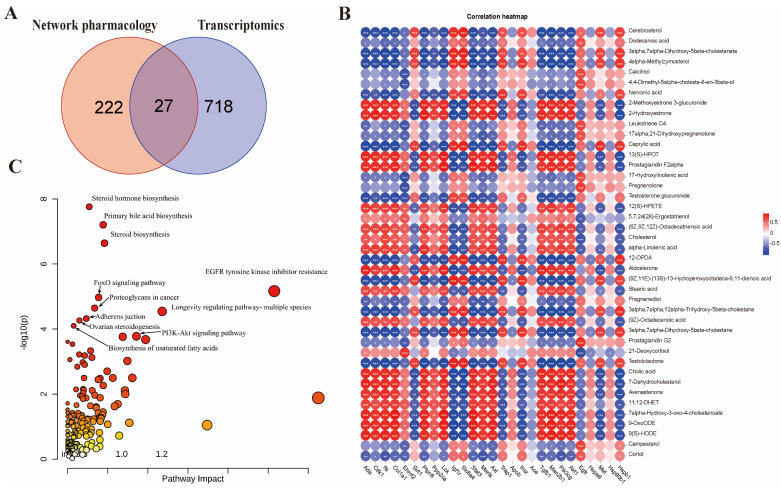
Multi-omics integrated analysis of LCW treatment for LN. (**A**) Common genes between network pharmacology and transcriptomics. (**B**) Spearman correlation heatmap of 27 overlapping genes and 42 candidate biomarkers. Color gradient indicates correlation strength (red: r > 0, positive association; blue: r < 0, negative association; *, **, and *** denote statistically significant differences (*p* < 0.05, *p* < 0.01, *p* < 0.001) between common genes and candidate biomarkers). (**C**) Joint pathway analysis of network pharmacology, transcriptomics, and metabolomics.

By integrating network pharmacology with transcriptomic data, 27 overlapping genes were successfully identified (Figure 6A). Figure 6B shows the correlation heatmap between 27 overlapping genes and 42 potential biomarkers. These genes were then combined with 42 candidate biomarkers and analyzed together using the MetaboAnalyst 6.0 platform (https://www.metaboanalyst.ca/) (accessed on 24 November 2024). The results (Figure 6C) revealed significant enrichment in various metabolic processes, including the generation of primary bile acids, steroid hormone biosynthesis, steroid production, formation of unsaturated fatty acids, as well as the FOXO and PI3K-AKT signaling pathways (*p* < 0.05).

### 2.7. LCW Inhibits Key Proteins in the PI3K/AKT/mTOR Pathway to Improve LN

**Figure 7:**
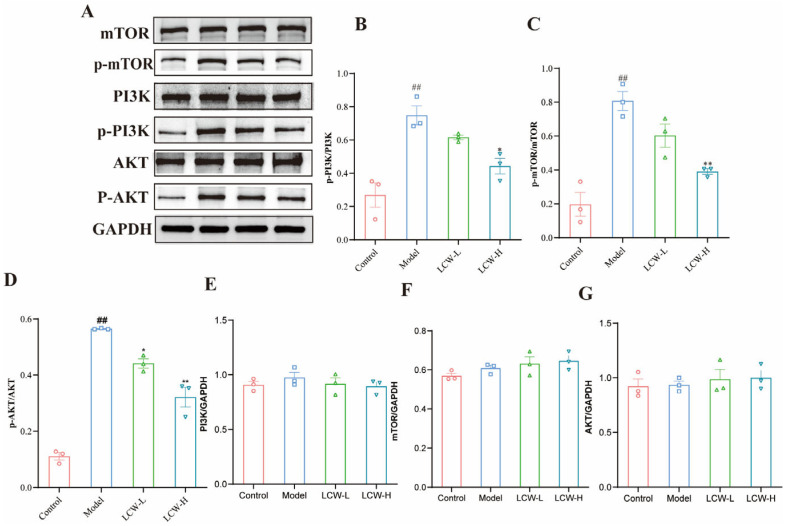
Western blot analysis of the expression of PI3K/AKT/mTOR signaling pathway-related proteins. (**A**) Individual protein bands and groupings. (**B**) p-PI3K protein expression. (**C**) p-AKT protein expression. (**D**) p-mTOR protein expression. (**E**) PI3K protein expression. (**F**) AKT protein expression. (**G**) mTOR protein expression. GAPDH was used as a loading control for grayscale analysis. Data are presented as mean ± standard error (*n* = 3). Compared to the control group, ^##^ *p* < 0.01; compared to the model group, * *p* < 0.05, ** *p* < 0.01.

The Western blot analysis results indicated that the levels of phosphorylated PI3K, phosphorylated AKT, and phosphorylated mTOR proteins were significantly higher in the experimental group compared to the control group (*p* < 0.01). Following LCW intervention, in the LCW-L group, there were no statistically significant changes in the expression of p-PI3K and p-mTOR when compared to the model group. However, the expression of p-AKT protein was significantly reduced (*p* < 0.05). In the LCW-H group, a significant down-regulation of p-PI3K, p-AKT, and p-mTOR protein expression was observed compared to the model group. (*p* < 0.05, *p* < 0.01, *p* < 0.01, respectively) (Figure 7A–D). Notably, the total PI3K, AKT, and mTOR expression remained unaltered in both groups (Figure 7E–G).

## 3. Discussion

LN is a kidney inflammation associated with SLE, and LCW is a traditional Chinese medicine preparation containing various bioactive components used in the treatment of LN. Although LCW has been used clinically, its mechanism of action remains unclear. This study utilized the MRL/lpr mouse model, along with UPLC-MS/MS, ELISA, histopathology, network pharmacology, transcriptomics, metabolomics, and Western blot techniques, to investigate the effective components of LCW in treating LN and its underlying mechanisms. This study is the first to elucidate the mechanisms by which LCW treats LN, providing further evidence for its clinical application, promotion, and the development of new drugs for treating LN.

### 3.1. Key Bioactive Components of LCW in LN Therapy

Initially, we identified 1303 active components in LCW, including quercetin, luteolin, and berberine, using UPLC-MS/MS technology. Studies have shown that quercetin and its derivatives can significantly suppress the production of pro-inflammatory cytokines, such as IL-6, IL-8, and TNF-α [17]. Additionally, quercetin can lower plasma concentrations of BUN and Cr, improving renal histopathological conditions in MRL/lpr mice [18]. Luteolin has been found to ameliorate pathological abnormalities and enhance renal function by reducing oxidative stress and UP levels in MRL/lpr mice [19]. Berberine exhibits anti-inflammatory and anti-renal fibrosis effects [20,21]. These properties suggest that the diverse bioactive constituents in LCW may play a substantial role in slowing the progression of LN.

### 3.2. LCW Ameliorates LN via Immune Regulation, Anti-Inflammatory Effects, and Renal Protection

To explore the potential mechanisms of action of LCW in the treatment of LN, a systematic pharmacodynamic evaluation was conducted using MRL/lpr mice [22]. ANA, as an initial indicator for screening LN, yield positive results, prompting further testing for anti-dsDNA to clarify the autoimmune response [23]. The titer of anti-dsDNA is closely associated with LN activity and serves as a key indicator for assessing disease status and monitoring therapeutic response [24]. Additionally, anti-Sm antibodies provide significant auxiliary diagnostic value [25]. Together, these three antibodies are essential markers of autoimmune activity in the diagnosis of LN. LCW treatment significantly reduced the levels of ANA, anti-dsDNA, and anti-Sm, suggesting that LCW can improve the pathological state of LN by inhibiting excessive autoimmune responses. Cr and BUN are primary indicators for assessing glomerular filtration function [26], while UP results from the destruction of the glomerular filtration barrier (GFB), which is a hallmark clinical manifestation of LN [27]. Therefore, Cr, BUN, and UP are critical markers of renal function impairment in LN. LCW treatment significantly improved the levels of Cr, BUN, and UP, indicating its substantial therapeutic effect in protecting renal function. The progression of LN is also closely linked to the abnormal levels of certain inflammatory cytokines. Studies have shown that TNF-α and IL-6 are involved in the pathogenesis of LN [28], promoting inflammatory responses and exacerbating kidney damage [29]. Moreover, IL-17 plays a dual role: it is both a disease-promoting cytokine in inflammatory conditions and an essential component of the immune system’s defense against infections [30]. IL-10, with its anti-inflammatory properties, may help restore immune balance [31]. LCW treatment reduced the levels of IL-6, IL-17, and TNF-α while increasing IL-10 levels, demonstrating its significant anti-inflammatory and immune-regulatory effects. Complement system components C3 and C4 are critical indicators of immune complex deposition and inflammatory responses [32]. LCW therapy notably increased C3 and C4 levels, suggesting its ability to inhibit excessive complement activation, which may contribute to improved management of LN.

Renal biopsy is considered the gold standard for diagnosing and predicting the prognosis of LN [33]. Inflammation is a hallmark of LN, making the control of glomerulonephritis a key therapeutic target. Fibrosis, a pathological process characterized by the continuous accumulation of connective tissue within the organ, leads to a reduction in parenchymal cells and damage to the organ’s structure and function. Organs that have sustained long-term damage and inflammation are particularly susceptible to fibrosis [34]. After LCW intervention, there was a significant reduction in inflammatory cell infiltration around the glomeruli in MRL/lpr mice. Additionally, interstitial fibrosis was alleviated, and IgG immune complex deposition decreased. These results suggest that LCW effectively improves renal lesions in MRL/lpr mice.

### 3.3. Network Pharmacology Reveals LCW’s Anti-LN Mechanisms

Network pharmacology analysis has identified key targets, such as the overexpression of TNF-α and IL-6, which exacerbate the symptoms of LN [35,36]. STAT3 has been proposed as a potential novel therapeutic target for LN [37]. Subsequent GO and KEGG analyses suggest that LCW may regulate gene expression and modulate the PI3K/Akt signaling pathway in the treatment of LN. Studies have shown that the activation of the PI3K/Akt/mTOR signaling pathway plays a crucial role in the pathogenesis of LN in mice [38]. These findings suggest that LCW may modulate the expression of factors such as TNF-α, IL-6, and STAT3, interfere with the PI3K/Akt signaling pathway, and exert anti-inflammatory and immune-modulatory effects, thereby slowing the progression of LN.

### 3.4. Transcriptomic Analysis

Transcriptomic analysis revealed that the GO and KEGG enrichment of DEGs was significantly associated with gene expression regulation, the FOXO signaling pathway, and the PI3K-AKT signaling pathway, consistent with predictions from network pharmacology. These findings suggest that LCW exerts its therapeutic effects on LN through multiple pathways and targets [10]. Previous studies have shown that FOXO3a plays a crucial role in inflammatory and autoimmune processes, and its upregulation alleviates symptoms by inhibiting the PI3K-AKT signaling pathway, a mechanism that helps reduce the severity of LN [39]. Additionally, FOXO1 overexpression significantly reduces glomerular cell proliferation and 24 h UP levels in MRL/lpr mice [40]. The PI3K/AKT/mTOR pathway is considered a key pathological feature of LN [41]. This pathway induces ribosome biogenesis, protein synthesis, cell growth, and the secretion of pro-inflammatory and mitotic factors, while inhibiting autophagy through the regulation of transcription factors such as FOXO [28]. These findings underscore the importance of targeting the PI3K/AKT/mTOR axis in the treatment of LN.

### 3.5. Metabolic Profiling Analysis

Metabolic profiling analysis identified 42 potential biomarkers closely linked to lipid metabolism. These biomarkers are primarily involved in lipid metabolic pathways such as steroid biosynthesis, steroid hormone biosynthesis, and arachidonic acid metabolism. This suggests that LCW-H may regulate key enzymes or metabolites in these pathways, influencing inflammatory responses and immune modulation, and subsequently correcting lipid metabolism disorders induced by LN. Existing studies have established that abnormal lipid metabolism is closely associated with the pathogenesis of LN [11], potentially disrupting lipid homeostasis in renal tissue and exacerbating glomerular and tubulointerstitial diseases [42]. Furthermore, disrupted lipid metabolism and its metabolites may stimulate the generation of IgG autoantibodies and cytokines, worsening the progression of LN. Importantly, the PI3K/AKT pathway plays a pivotal role in lipid metabolism regulation [43]. Based on these findings, we hypothesize that LCW-H may regulate lipid metabolism through the PI3K/AKT pathway to exert therapeutic effects on LN.

### 3.6. Integrated Multi-Omics Analysis

The integrated analysis using network pharmacology, transcriptomics, and metabolomics has revealed significant enrichment in pathways such as primary bile acid biosynthesis, the FOXO signaling pathway, and the PI3K-AKT signaling pathway in the treatment of LN with LCW. Notably, the PI3K/AKT signaling pathway plays a pivotal role in regulating metabolism, inflammation, and immune responses in immune cells [44]. In LN, the abnormal activation of the PI3K-AKT pathway and its downstream mTOR signaling pathway is directly linked to inflammation, immune dysregulation, and renal fibrosis. This pathway promotes the proliferation and activation of renal fibroblasts, which are key contributors to kidney injury [39,45]. Therefore, future studies will focus on elucidating the specific mechanisms by which LCW modulates the PI3K/AKT/mTOR signaling pathway in the treatment of LN.

### 3.7. LCW Ameliorates LN via Suppression of PI3K/AKT/mTOR Signaling

The PI3K pathway functions through its regulatory p85 and catalytic p110 subunits, which activate the AKT/PKB effector downstream. AKT exists in three isoforms AKT1, AKT2, and AKT3 each playing crucial roles in cell signaling. mTOR, a serine/threonine kinase, governs cellular metabolism and is vital for autophagy [46]. As a downstream target of the PI3K/AKT pathway, mTOR acts as a negative regulator of autophagy by inhibiting the UNC-51-like autophagy-activating kinase (ULK) complex and preventing the formation of autophagosomes [47]. Disruption of the autophagy exacerbates inflammatory cytokine production and impairs apoptotic cell clearance, both of which contribute to the progression of LN [48]. We employed Western blot to assess key proteins within the PI3K/AKT/mTOR signaling pathway. The results revealed significant upregulation of p-PI3K, p-AKT, and p-mTOR in kidney tissue from MRL/lpr mice, consistent with prior studies [46,49]. Increased levels of p-p85 (PI3K) suggest that its activation could be a pivotal driver of aberrant renal cell proliferation and survival. Activation of PI3K leads to the conversion of PIP2 to PIP3 on the cell membrane, recruiting AKT to the membrane and promoting its phosphorylation to p-AKT [50]. As a central molecule in this signaling cascade, p-AKT regulates cell survival and growth by phosphorylating downstream target proteins. mTOR is subsequently activated through p-AKT, and its aberrant activation disrupts immune balance, promotes autoimmune responses, induces renal fibrosis, inhibits autophagy, and contributes to kidney damage [51]. The PI3K/AKT/mTOR pathway is highly activated in MRL/lpr mice, underscoring its critical role in LN pathogenesis [45]. Following LCW treatment, we observed a significant reduction in p-AKT expression in the LCW-L group, although changes in p-PI3K and p-mTOR levels were not statistically significant. In contrast, in the LCW-H group, p-PI3K, p-AKT, and p-mTOR expression were all notably downregulated, suggesting that LCW-H effectively inhibits the abnormal activation of the PI3K/AKT/mTOR pathway. In addition, previous studies have shown that berberine can downregulate the PI3K/AKT pathway, thereby reducing apoptosis of renal tubular epithelial cells [52]. It can also inhibit mTOR, influencing energy metabolism and promoting apoptosis [53]. Luteolin and quercetin have been found to inhibit the activation of the PI3K/AKT signaling pathway and the formation of renal fibrosis [54,55,56]. This inhibitory effect may play a crucial protective role by promoting renal cell autophagy and reducing apoptosis, thereby slowing the progression of kidney damage. These findings imply that LCW may treat LN through the inhibition of the PI3K/AKT/mTOR signaling pathway, offering a potential strategy for managing fibrosis associated with LN and regulating cellular autophagy processes.

### 3.8. Limitations and Prospects

The present study employs the MRL/lpr mouse model to simulate LN, which is widely used in LN research. However, compared to human LN, there are still differences in its pathophysiological processes and immune responses. Therefore, future research needs to be conducted in models that are closer to human LN to further validate the therapeutic effects and mechanisms of LCW. Meanwhile, omics data primarily reflect changes in biological processes and cannot directly prove causality. Therefore, more in-depth experimental validations, such as gene knockout and signal pathway inhibitors, are required to clarify the targets and mechanisms of LCW’s action. Despite these limitations, this study provides new insights and evidence for LCW’s treatment of LN. Future research can further explore the active components and targets of LCW and conduct clinical trials to potentially offer new effective treatments for LN.

In summary, this study has revealed that the active components in LCW, including quercetin, luteolin, and berberine, play a significant role in exerting anti-inflammatory and immunomodulatory effects, while also improving renal function in MRL/lpr mice. Furthermore, our findings demonstrate that LCW-H exerts its protective effects by inhibiting the abnormal activation of the PI3K/AKT/mTOR signaling pathway. However, to fully elucidate the precise mechanisms underlying LCW’s therapeutic actions, further research is required.

## 4. Materials and Methods

### 4.1. Drugs and Reagents

Lang Chuang Wan (LCW, batch number National Medicine Approval Z22021973) was provided by Changchun Haiwai Pharmaceutical Group Co., Ltd. (Changchun, China), with a dosage of 0.5 g per tablet. Prednisone Acetate Tablets (PA, National Medicine Approval H12020689) were supplied by Tianjin Tianyao Pharmaceutical Co., Ltd. (Tianjin, China), with a dosage of 5 mg per tablet.

### 4.2. UPLC–MS/MS Analysis of LCW Components

Accurately weigh 15.6 mg of LCW powder and transfer it into a 2 mL centrifuge tube. Add 600 µL of methanol containing 2-amino-3-(2-chloro-phenyl)-propionic acid (at 4 ppm). Vortex for 30 s, then add steel balls and place the tube in a tissue grinder. Grind for 60 s at 55 Hz. Perform ultrasound treatment at room temperature for 15 min, followed by centrifugation at 12,000 rpm and 4 °C for 10 min. Filter the supernatant through a 0.22 µm membrane and transfer it to a detection vial for UPLC-MS/MS analysis [57].

The analysis was conducted using a Vanquish UHPLC System (Thermo Fisher Scientific, Waltham, MA, USA) for liquid chromatography. Chromatographic separation was achieved with an ACQUITY UPLC^®^ HSS T3 column (2.1 × 100 mm, 1.8 µm) (Waters, Milford, MA, USA), maintained at a consistent temperature of 40 °C. The flow rate and injection volume were set at 0.3 mL/min and 2 μL, respectively. For LC-ESI (+)-MS analysis, the mobile phases consisted of 0.1% formic acid in acetonitrile (*v*/*v*) (B2) and 0.1% formic acid in water (*v*/*v*) (A2). The chromatographic gradient was as follows: 0–1 min, 8% B2; 1–8 min, 8% to 98% B2; 8–10 min, 98% B2; 10–10.1 min, 98% to 8% B2; 10.1–12 min, and 8% B2. For LC-ESI (−)-MS analysis, acetonitrile (B3) and 5 mM ammonium formate (A3) were used as the mobile phases. The gradient for this analysis was as follows: 0–1 min, 8% B3; 1–8 min, 8% to 98% B3; 8–10 min, 98% B3; 10–10.1 min, 98% to 8% B3; 10.1–12 min, and 8% B3 [58]. Metabolite detection was carried out using a Q Exactive mass spectrometer (Thermo Fisher Scientific, USA), equipped with an ESI ion source. Both MS1 and MS/MS data were obtained in Full MS-ddMS2 mode (data-dependent MS/MS). The instrument parameters were as follows: sheath gas pressure of 40 arb, auxiliary gas flow of 10 arb, spray voltages of 3.50 kV (ESI+) and −2.50 kV (ESI−), capillary temperature of 325 °C, MS1 scan range of *m*/*z* 100–1000, MS1 resolving power of 70,000 FWHM, 10 data-dependent scans per cycle, MS/MS resolving power of 17,500 FWHM, normalized collision energy of 30 eV, and automatic dynamic exclusion [59].

### 4.3. Animal and Experimental Design

Eight-week-old female BALB/c and MRL/lpr mice, each with a body weight of 20 ± 2 g, were obtained from Spero Biotech Co., Ltd. (Beijing, China), under the animal license number SCXK (Jing) 2019-0010. The mice were housed in a standard specific pathogen-free (SPF) environment, maintained on a 12 h light/dark cycle, with free access to both food and water. The study was conducted in accordance with ethical guidelines, and the protocol was approved by the Ethics Committee of Changchun University of Chinese Medicine (approval number: 2023513).

After a one-week acclimatization period, six BALB/c mice were assigned to the control group (control), and 24 MRL/lpr mice were randomly divided into four groups (*n* = 6) based on a random number table: the model group (model), the positive control group (PA, 5.0 mg/kg), the low-dose Lang Chuang Wan group (LCW-L, 1.6 g/kg), and the high-dose Lang Chuang Wan group (LCW-H, 3.2 g/kg). Both the control and model groups were administered the same volume of normal saline via gavage. All mice were treated daily via gavage for 8 weeks. Urine samples were collected at weeks 0, 2, 4, 6, and 8. At the conclusion of the study, all mice were euthanized, and blood samples along with kidney tissue were collected. Blood samples were centrifuged at 3000 rpm for 10 min at 4 °C to obtain serum, which was used for ELISA and serum metabolomics analyses. Kidney tissue was rapidly excised and stored as follows: portions of the tissue were immediately frozen in liquid nitrogen and stored at −80 °C for subsequent transcriptomics and Western blot analyses, while the remaining tissue was fixed in 4% paraformaldehyde for histopathological examination.

### 4.4. ELISA Analysis

The serum levels of ANA, anti-dsDNA, anti-Sm antibodies (anti-Sm), complement components C3 and C4, serum creatinine (Cr), blood urea nitrogen (BUN), UP, IL-6, IL-10, IL-17, and tumor necrosis factor-α (TNF-α) in mice were measured using an ELISA kit (Jiangsu Meimian Industrial Co., Ltd., Yancheng, China). All procedures were carried out in accordance with the manufacturer’s guidelines.

### 4.5. Histopathological Analysis of Kidney Tissue

Kidney tissues were fixed in 4% paraformaldehyde, then subjected to a dehydration process through an alcohol gradient before being immersed in wax. After being impregnated with wax, the tissues were embedded, frozen, and trimmed. The cooled wax blocks were then placed in a paraffin sectioning machine, where 4 μm thick sections were sliced and mounted onto slides. These sections were subsequently stained with hematoxylin and eosin (H&E) and Masson’s trichrome stains. After staining, the sections were scanned and analyzed for renal histopathological features.

For dewaxing and rehydration, xylene and a series of alcohol gradients were used. Antigen retrieval was carried out using an EDTA-based retrieval buffer. Following a 30 min blocking step with BSA, the sections were incubated overnight at 4 °C with the primary antibody. Following this, the samples were washed three times with PBS for 5 min each. The corresponding secondary antibody was applied and incubated at room temperature for 1 h. Cell nuclei were counterstained with DAPI, and the slides were mounted with an anti-fluorescence quencher before being scanned or photographed for detailed observation.

### 4.6. Network Pharmacology

The active components and targets of LCW were obtained from the Traditional Chinese Medicine Systems Pharmacology Database and Analysis Platform (TCMSP, https://www.tcmsp-e.com/load_intro.php?id=43 (accessed on 15 July 2024), using oral bioavailability (OB) >30% and drug-likeness (DL) ≥0.18 as selection criteria. Additional target information was collected from SwissTargetPrediction (http://www.swisstargetprediction.ch/ (accessed on 15 July 2024)), PubChem (https://pubchem.ncbi.nlm.nih.gov/ (accessed on 15 July 2024)), and relevant literature [10], with a screening threshold of probability > 0. Targets related to LN were obtained from the OMIM database (https://www.omim.org/), GeneCards (http://www.genecards.org/), and DrugBank (https://go.drugbank.com/) using “lupus nephritis” as the search term. The LCW targets were then mapped to LN-related targets using the Bioinformatics website (https://www.bioinformatics.com.cn/), and intersection targets were identified, representing the genes involved in the treatment of LN by LCW. The intersection targets were subsequently uploaded to the STRING 12.0 database (https://cn.string-db.org/) and analyzed using Cytoscape 3.7.2 software to construct a protein–protein interaction (PPI) network, with a high-confidence threshold of ≥0.7. Core targets were identified based on degree centrality, betweenness centrality, and closeness centrality. Finally, the intersection targets were subjected to Gene Ontology (GO) and Kyoto Encyclopedia of Genes and Genomes (KEGG) pathway enrichment analysis using the DAVID tool (https://david.ncifcrf.gov/).

### 4.7. Kidney Tissue Transcriptomic Analysis

RNA was isolated from animal tissues using the TRIzol Reagent, following the protocol provided in the instruction manual. The reagent was supplied by Life Technologies, Carlsbad, California, USA. The RNA yield and purity were quantified using the NanoDrop 2000, an instrument from Thermo Fisher Scientific (Wilmington, DE, USA). RNA quality was assessed by evaluating its integrity with the RNA Nano 6000 Assay Kit, which was analyzed on the Agilent Bioanalyzer 2100 system, provided by Agilent Technologies (Santa Clara, CA, USA). This meticulous process ensured the RNA’s integrity for subsequent experimental procedures. The libraries were sequenced on an Illumina NovaSeq platform to generate 150 bp paired-end reads, in accordance with the manufacturer’s guidelines. The raw reads were then processed using the BMK Cloud bioinformatics pipeline (www.biocloud.net (accessed on 12 February 2025)). Initially, the raw data in fastq format were filtered using in-house Perl scripts to obtain clean reads by removing adapter sequences, reads containing poly-N, and low-quality sequences. During this step, key quality metrics such as Q20, Q30, GC content, and sequence duplication levels were calculated. All subsequent analyses were based on high-quality clean data. After processing, adapter sequences and low-quality reads were removed, resulting in clean reads. The clean reads were subsequently aligned to the reference genome using the Hisat2 tool. Only those reads with a perfect match, or a single mismatch, were considered for further analysis and annotation. Gene expression levels were quantified by calculating fragments per kilobase of transcript per million fragments mapped (FPKM), with the formula: FPKM = cDNA Fragments/(Mapped Fragments (Millions) × Transcript Length (kb). Differential expression analysis between two conditions was performed using DESeq2. DESeq2 provides statistical routines for determining differential expression in digital gene expression data using a model based on the negative binomial distribution. Differentially Expressed Genes (DEGs) were filtered using the criteria of FDR < 0.05 and |log2Fold Change| > 1. Subsequently, visual enrichment analysis of these DEGs was performed using the DAVID database, and weishengxin.

### 4.8. Serum Metabolomic Analysis

The metabolomics analysis was performed using a state-of-the-art LC/MS system, featuring a Waters Acquity I-Class PLUS UPLC, coupled with the high-definition Waters Xevo G2-XS QTof mass spectrometer. Chromatographic separation was performed using a Waters Acquity UPLC HSS T3 column (1.8 µm, 2.1 × 100 mm). The mobile phases for both positive and negative ion modes included 0.1% formic acid in water (phase A) and 0.1% formic acid in acetonitrile (phase B), with an injection volume of 2 μL. The Xevo G2-XS QTof mass spectrometer was operated in MSe mode, enabling simultaneous collection of full and fragmented mass spectra, as controlled by MassLynx V4.2 software. The system supported dual-channel acquisition with a collision energy range of 10 to 40 volts and a scan rate of 0.2 s per spectrum. The ESI ion source settings included a capillary voltage of 2500 V for positive ions and −2000 V for negative ions, a cone voltage of 30 V, and an ion source temperature of 100 °C. Desolvation gas was set to 500 °C with flow rates of 50 L/h for backflush and 800 L/h for desolvation. Raw data were collected using MassLynx V4.2 and processed with Progenesis QI software (version 4.0) for peak detection, alignment, and subsequent refinement. Compound identification was supported by the METLIN database and Biomark’s library. After normalization against the total peak area, principal component analysis (PCA) and Spearman correlation analysis were performed to assess the consistency between the samples and QC. Metabolite characterization was further conducted using the KEGG, HMDB, and Lipid Maps databases. Differential metabolites were identified through a *t*-test for significance (*p* < 0.05), and orthogonal partial least squares discriminant analysis (OPLS-DA) was performed using the “ropls” R package 4.5.1, supported by 200 permutation tests for validation. Selection of key metabolites was based on variable importance in projection (VIP) values and *p*-values. KEGG pathway enrichment analysis for these metabolites was conducted using a hypergeometric test (*p* < 0.05).

### 4.9. Western Blotting

The expression levels of p-PI3K, PI3K (phosphatidylinositol 3-kinase), p-AKT, AKT (protein kinase B), p-mTOR, and mTOR (mammalian target of rapamycin) in mouse kidney tissue were assessed by Western blot. The primary antibody reagents used were as follows: p-AKT (80455-1-RR), mTOR (28273-1-AP), and GAPDH (60004-1-Ig) from Wuhan Sanying Biotechnology Co., Ltd. (Wuhan, China); PI3K (AF6241) and p-PI3K (AF3241) from Affinity Biosciences Ltd. (Liyang, Jiangsu, China); AKT (A18675) from ABclonal Biotechnology Co., Ltd. (Wuhan, China); and p-mTOR (5536S) from Cell Signaling Technology, Inc. (Danvers, MA, USA).

Kidney tissue samples were carefully minced and homogenized in RIPA lysis buffer to denature the proteins. The homogenate was vortexed and centrifuged at 12,000 rpm for 10 min at 4 °C to collect the supernatant. Protein concentration was quantified using the BCA assay, and the protein supernatant was stored at −20 °C until further use. Sodium dodecyl sulfate-polyacrylamide gel electrophoresis (SDS-PAGE) was conducted, followed by membrane transfer and blocking. The membrane was then incubated overnight at 4 °C with primary antibodies against PI3K, p-PI3K, AKT, p-mTOR (1:1000 dilution), mTOR (1:5000 dilution), p-AKT (1:30,000 dilution), and GAPDH (1:50,000 dilution). Afterward, the membrane was incubated with HRP-conjugated secondary antibodies (1:10,000 dilution) at room temperature for 2 h. The membrane was subsequently washed, and protein bands were visualized using an ECL reagent and a chemiluminescence imaging system (Hangzhou Shenhua Technology Co., Ltd., SH-523, Hangzhou, China). Images were then captured and analyzed with ImageJ software, version 1.53a.

### 4.10. Statistical Analysis

All data are expressed as mean ± standard error of the mean (SEM). Statistical analyses were performed using GraphPad Prism 9.5 software (GraphPad Software, Inc., Boston, MA, USA). Group differences were assessed using one-way analysis of variance (ANOVA), with subsequent data visualization in the form of charts. Statistical significance was considered at a *p*-value of less than 0.05.

## 5. Conclusions

This study demonstrates that LCW exhibits significant pharmacological effects in MRL/lpr mice, particularly by inhibiting the PI3K/AKT/mTOR signaling pathway, thereby effectively alleviating the symptoms of LN. These findings provide strong theoretical support for the clinical application of LCW, and the development of new therapeutic agents based on its active components.

## Figures and Tables

**Table 1:** The 25 Active Constituents in LCW.

No.	Compounds	Formula	Precursor Type	Exact Mass	Class	Peak Area
1	Berberine	[C_20_H_18_NO_4_]+	[M + H]+	336.12358	Alkaloids	765,955,338
2	Chrysin	C_15_H_10_O_4_	[M − H]−	254.05791	Flavonoids	959,729,637.3
3	Licoisoflavone a	C_20_H_18_O_6_	[M + H]+	354.11033	Flavonoids	343,842,417.9
4	Desmethylxanthohumol	C_20_H_20_O_5_	[M + H]+	340.13107	Flavonoids	234,716,721.9
5	Rutin	C_27_H_30_O_16_	[M − H]−	610.15338	Flavonoids	192,697,182
6	2,4,6-trihydroxydihydrochalcone	C_15_H_14_O_4_	[M − H]−	258.0892	Flavonoids	73,232,107.88
7	Baicalein	C_15_H_10_O_5_	[M + H]+	270.05282	Flavonoids	28,482,364.63
8	2′,7-dihydroxy-4′,5′-dimethoxyisoflavone	C_17_H_14_O_6_	[M − H]−	314.07903	Flavonoids	18,475,453.99
9	Daidzin	C_21_H_20_O_9_	[M − H_2_O − H]−	416.11073	Flavonoids	7,967,367.338
10	Acacetin	C_16_H_12_O_5_	[M + H]+	284.06847	Flavonoids	6,688,437.089
11	Luteolin	C_15_H_10_O_6_	[M − H_2_O + H]+	286.04774	Flavonoids	4,144,178.487
12	Tibolone	C_21_H_28_O_2_	[M − H]−	312.20892	Hormone class	24,691,286.41
13	Syringic acid	C_9_H_10_O_5_	[M − H]−	198.05282	Phenolic acids	1,025,933,162
14	3-o-feruloylquinic acid	C_17_H_20_O_9_	[M − H]−	368.11073	Phenolic acids	353,099,163.3
15	5-caffeoylquinic acid	C_16_H_18_O_9_	[M − H]−	354.09508	Phenolic acids	272,904,467.6
16	5-feruloylquinic acid	C_17_H_20_O_9_	[M − H]−	368.11073	Phenolic acids	177,445,299.3
17	Quinic acid	C_7_H_12_O_6_	[M − H]−	192.06339	Phenolic acids	112,171,658.5
18	Caffeic acid	C_9_H_8_O_4_	[M − H_2_O + H]+	180.04226	Phenolic acids	21,905,035.5
19	Beta-sitosterol	C_29_H_50_O	[M − H_2_O + H]+	414.38614	phytosterols	41,671,858.23
20	Oleanoic acid	C_30_H_48_O_3_	[M − H]−	456.36033	Terpenoids	2,895,688,889
21	Hederagenin	C_30_H_48_O_4_	[M − H]−	472.35524	Terpenoids	1,355,770,073
22	Glycyrrhetinic acid	C_30_H_46_O_4_	[M + H]+	470.33959	Terpenoids	379,591,568.6
23	Albiflorin	C_23_H_28_O_11_	[M + H]+	480.16315	Terpenoids	178,862,591.3
24	Licoricesaponin g2	C_42_H_62_O_17_	[M + H]+	838.39868	Terpenoids	27,520,043.37
25	Dihydrotanshinone i	C_18_H_14_O_3_	[M − H]−	278.09429	Terpenoids	15,131,118.85

**Table 2:** Differential metabolites in MRL/lpr mouse serum between groups.

No.	Metabolite Name	Molecular Formula	HMDB ID	Model/Control	LCW-H/Model
1	Cerebrosterol	C_27_H_46_O_2_	HMDB0001419	↓	↑
2	Dodecanoic acid	C_12_H_24_O_2_	HMDB0000638	↓	↑
3	3alpha,7alpha-Dihydroxy-5beta-cholestanate	C_27_H_46_O_4_	HMDB0000359	↓	↑
4	4alpha-Methylzymosterol	C_28_H_46_O	HMDB0001217	↓	↑
5	Calcitriol	C_27_H_44_O_3_	HMDB0001903	↓	↑
6	4,4-Dimethyl-5alpha-cholesta-8-en-3beta-ol	C_29_H_50_O	HMDB0006840	↓	↑
7	Nervonic acid	C_24_H_46_O_2_	HMDB0002368	↓	↑
8	2-Methoxyestrone 3-glucuronide	C_25_H_32_O_9_	HMDB0004482	↑	↓
9	2-Hydroxyestrone	C_18_H_22_O_3_	HMDB0000343	↑	↓
10	Leukotriene C4	C_30_H_47_N_3_O_9_S	HMDB0001198	↓	↑
11	17alpha,21-Dihydroxypregnenolone	C_21_H_32_O_4_	HMDB0006762	↓	↑
12	Caprylic acid	C_8_H_16_O_2_	HMDB0000482	↓	↑
13	13(S)-HPOT	C_18_H_30_O_4_	HMDB0301803	↑	↓
14	Prostaglandin F2alpha	C_20_H_34_O_5_	HMDB0001139	↑	↓
15	17-Hydroxylinolenic acid	C_18_H_30_O_3_	HMDB0011108	↓	↑
16	Pregnenolone	C_21_H_32_O_2_	HMDB0000253	↓	↑
17	Testosterone glucuronide	C_25_H_36_O_8_	HMDB0003193	↓	↑
18	12(S)-HPETE	C_20_H_32_O_4_	HMDB0004243	↑	↓
19	5,7,24(28)-Ergostatrienol	C_28_H_44_O	HMDB0060404	↑	↓
20	(6Z,9Z,12Z)-Octadecatrienoic acid	C_18_H_30_O_2_	HMDB0003073	↓	↑
21	Cholesterol	C_27_H_46_O	HMDB0000067	↑	↓
22	alpha-Linolenic acid	C_18_H_30_O_2_	HMDB0001388	↑	↓
23	12-OPDA	C_18_H_28_O_3_	HMDB0301804	↓	↑
24	Aldosterone	C_21_H_28_O_5_	HMDB0000037	↑	↓
25	(9Z,11E)-(13S)-13-Hydroperoxyoctadeca-9,11-dienoic acid	C_18_H_32_O_4_	HMDB0003871	↓	↑
26	Stearic acid	C_18_H_36_O_2_	HMDB0000827	↑	↓
27	Pregnanediol	C_21_H_36_O_2_	HMDB0004025	↓	↑
28	3alpha,7alpha,12alpha-Trihydroxy-5beta-cholestane	C_27_H_48_O_3_	HMDB0001457	↓	↑
29	(9Z)-Octadecenoic acid	C_18_H_34_O_2_	HMDB0000207	↑	↓
30	3alpha,7alpha-Dihydroxy-5beta-cholestane	C_27_H_48_O_2_	HMDB0006893	↓	↑
31	Prostaglandin G2	C_20_H_32_O_6_	HMDB0003235	↓	↑
32	21-Deoxycortisol	C_21_H_30_O_4_	HMDB0004030	↓	↑
33	Testololactone	C_19_H_26_O_3_	HMDB0258855	↓	↑
34	Cholic acid	C_24_H_40_O_5_	HMDB0000619	↑	↑
35	7-Dehydrocholesterol	C_27_H_44_O	HMDB0000032	↑	↓
36	Avenastenone	C_29_H_46_O	HMDB0304267	↑	↓
37	11,12-DHET	C_20_H_34_O_4_	HMDB0002314	↑	↓
38	7alpha-Hydroxy-3-oxo-4-cholestenoate	C_27_H_42_O_4_	HMDB0012458	↑	↓
39	9-OxoODE	C_18_H_30_O_3_	HMDB0004669	↑	↓
40	9(S)-HODE	C_18_H_32_O_3_	HMDB0004670	↑	↓
41	Campesterol	C_28_H_48_O	HMDB0002869	↓	↑
42	Cortol	C_21_H_36_O_5_	HMDB0003180	↓	↑

↑ indicates upregulation of differential metabolites when comparing Model vs. Control or LCW-H vs. Model. ↓ indicates downregulation of differential metabolites when comparing Model vs. Control or LCW-H vs. Model.

## Data Availability

The original contributions presented in this study are included in the article. Further inquiries can be directed to the corresponding authors.

## Abbreviations

The following abbreviations are used in this manuscript:

SLE	Systemic lupus erythematosus
LN	Lupus nephritis
LCW	Lang Chuang Wan
ANA	Anti-nuclear antibodies
ANOVA	One-way analysis of variance
anti-dsDNA	Anti-double-stranded DNA antibodies
anti-Sm	Anti-Sm antibody
BP	Biological processes
CC	Cellular components
MF	Molecular functions
GO	Gene Ontology
KEGG	Kyoto Encyclopedia of Genes and Genomes
BPC	Base peak chromatograms
BUN	Blood urea nitrogen
DAVID	Database for annotation, visualization and integrated discovery
DEGs	Differentially expressed genes
DEM	Differentially expressed metabolites
ELISA	Enzyme-linked immunosorbent assay
HMDB	Human metabolome database
OPLS-DA	Orthogonal partial least squares discriminant analysis
PCA	Principal component analysis
SEM	Standard error of the mean
UPLC-MS/MS	Ultra-performance liquid chromatography–tandem mass spectrometry
UP	Urinary protein
VIP	Variable importance for the projection
